# Low ambient temperature during early postnatal development fails to cause a permanent induction of brown adipocytes

**DOI:** 10.1096/fj.15-271395

**Published:** 2015-04-20

**Authors:** Agnieszka Chabowska-Kita, Anna Trabczynska, Agnieszka Korytko, Monika M. Kaczmarek, Leslie P. Kozak

**Affiliations:** Institute of Animal Reproduction and Food Research, Polish Academy of Sciences, Olsztyn, Poland

**Keywords:** sympathetic activity, diet-induced obesity, recombinant inbred mice, brown adipocyte involution, microarray gene expression

## Abstract

The brown adipocyte phenotype (BAP) in white adipose tissue (WAT) is transiently induced in adult mammals in response to reduced ambient temperature. Since it is unknown whether a cold challenge can permanently induce brown adipocytes (BAs), we reared C57BL/6J (B6) and AxB8/PgJ (AxB8) mice at 17 or 29°C from birth to weaning, to assess the BAP in young and adult mice. Energy balance measurements showed that 17°C reduced fat mass in the preweaning mice by increasing energy expenditure and suppressed diet-induced obesity in adults. Microarray analysis of global gene expression of inguinal fat (ING) from 10-day-old (D) mice indicates that expression at 17°C *vs.* 29°C was not different. Between 10 and 21 days of age, the BAP was induced coincident with morphologic remodeling of ING and marked changes in expression of neural development genes (*e.g.*, *Akap 12* and *Ngfr*). Analyses of *Ucp1* mRNA and protein showed that 17°C transiently increased the BAP in ING from 21D mice; however, BAs were unexpectedly present in mice reared at 29°C. The involution of the BAP in WAT occurred after weaning in mice reared at 23°C. Therefore, the capacity to stimulate thermogenically competent BAs in WAT is set by a temperature-independent, genetically controlled program between birth and weaning.—Chabowska-Kita, A., Trabczynska, A., Korytko, A., Kaczmarek, M. M., Kozak, L. P. Low ambient temperature during early postnatal development fails to cause a permanent induction of brown adipocytes.

Mammals possess 2 distinct forms of adipose tissue serving opposite functions in the regulation of energy balance. White adipose tissue (WAT) synthesizes and stores excess energy in the form of triacylglycerols, whereas brown adipose tissue (BAT) specializes in dissipation of chemical energy to produce heat for defense against cold. Interscapular (i)BAT in mice appears first during the final days of fetal development, reaches peak values in the first days after birth, and remains relatively constant during life (1, 2). In contrast, brown adipocytes (BAs) in traditional white fat [brite; (3)] are induced at an ambient temperature of 23°C at approximately 21 days of age, disappear spontaneously by 35 days of age (2), but can reemerge when the animal is exposed to cold or treated with a β3-adrenergic agonist (4–7). Subcutaneous WAT in mice develops relatively late in embryogenesis, with adipocyte lipid components already evident on postnatal day 1, whereas visceral WAT precursor cells appear first at postnatal day 4, with lipid accumulation occurring at 7 days of age (8).

Perturbation of energy balance during early development by dietary manipulations causes cellular, molecular, and biochemical adaptations that affect physiology and metabolism in adulthood (9). Undernutrition during the lactation period causes a major negative energy balance, evidenced in hypoglycemia, hypoinsulinemia and hypoleptinemia, reduced adiposity, and suppression of the brown adipocyte phenotype (BAP) (10–12). An obvious alternative to perturbing energy balance in the neonatal pup through reduced energy intake is to increase energy expenditure (EE) by reducing ambient temperature. In addition to changes in energy balance when neonatal pups are raised at reduced ambient temperature, qualitative and quantitative changes may be induced in BAT or other potential sites of thermogenesis, such as skeletal muscle (13). The amount of iBAT may be increased, and the differentiation of brite cells in WAT may occur precociously or at higher levels as a result of the challenge of a cooler environment. Morrison *et al.* (14) found that rats reared at 18°C from birth to 60 days of age had increased norepinephrine content in iBAT and an increase in the number of sympathetic ganglion cells projecting to iBAT when assayed 30 days after being returned to an ambient temperature of 23°C. This study provides compelling evidence for permanent changes in sympathetic activity in the iBAT of adult rats reared in a cold environment that could have an impact on the development of obesity. The role of sympathetic nervous system (SNS) innervation in BAT proliferation and thermogenesis was first described 3 decades ago (15) and it has been studied extensively since then (16). Physiologic and neuroanatomic evidence for SNS innervation of WAT in mammals suggests its role in the regulation of total body fat (17), initiating WAT lipid mobilization (18), and regulating the number of fat cells (19).

Accordingly, we predicted that decreased ambient temperature during early postnatal development would determine the capacity for BA induction in a manner that would reduce susceptibility to diet-induced obesity (DIO) in adulthood. We therefore designed an experiment in which dams and their newborn litters were maintained at cold (17°C) or thermoneutral (29°C) ambient temperatures from birth until weaning at 21 days of age. We compared C57BL/6J (B6) mice to AxB8/PgJ (AxB8) recombinant inbred mice, the latter characterized by higher induction of brite cells within white fat depots upon adrenergic stimulation (6, 20). Low ambient temperature during early postnatal development reduced fat mass (FM) and adiposity in developing and adult mice fed a high-fat diet (HFD) for 8 weeks. The results indicate that the development of BAs in WAT and their thermogenic potential is determined by a genetic program that is independent of ambient temperature. The realization of the complete BAP in brite cells during the early postnatal period in response to cold stimulation, a process that is recapitulated in adult animals, depends on the development of sympathetic responsiveness to low ambient temperatures in WAT between 10 and 21 days of age.

## MATERIALS AND METHODS

### Animals

B6 and recombinant inbred AxB8 mice, purchased from The Jackson Laboratory (Bar Harbor, ME, USA), were bred at the Institute of Animal Reproduction and Food Research of the Polish Academy of Sciences. Breeders and newborn mice (8 pups per litter) were maintained at 17 or 29°C in a 12-hour light-dark cycle. The ambient temperature during the lactation period from birth to 21 days of age had no effect on the survival of the pups. Of 844 mice in the experiment, only 20 died, and those were equally distributed among strains and ambient temperatures. After weaning, the offspring mice were moved to 23°C in group housing (2–4 mice per pen) and fed a standard chow diet (STD;13 kcal% fat; PicoLab Rodent Diet 20 5053; Columbia, MO, USA) *ad libitum* until 8 weeks of age. From 56 days of age, the mice were housed individually. They were fed an HFD (59 kcal% fat; TestDiet AIN-76A; LabDiet) *ad libitum* for 8 weeks or were fed an STD and exposed to 4°C for 7 days. The 112D mice were anesthetized for blood collection, and, for tissue collection, were killed by decapitation or cervical dislocation. Tissues were immediately frozen in liquid nitrogen and stored at −80°C for subsequent analysis. All experiments were approved by the local ethics committee of the University of Warmia and Mazury.

### Phenotypic assays

#### Body composition

Adiposity was determined from body weight (BW) and body composition measurements performed by NMR (Bruker, Billerica, MA, USA) at days 7, 10, 15, 21, 56, and 112 of postnatal development.

#### Quantitative RT-PCR

Total RNA was isolated from adipose tissue with TRI Reagent and BCP phase separation reagent (both from Molecular Research Center, Inc., Cincinnati, OH, USA). RNA was purified with the RNeasy Mini Kit (Qiagen, Valencia, CA, USA) and stored at −80°C in Ambion RNase-free H_2_O with an addition of SUPERase-In (Thermo Fisher Scientific, Waltham, MA, USA) for RNA protection. Quality and quantity of RNA were determined by UV spectrophotometry (Nanodrop; Thermo Fisher Scientific) and agarose gel visualization. Quantitative RT-PCR with TaqMan probes and primers (Life-Technologies-Applied Biosystems, Foster City, CA, USA) was performed with standard curves generated from pooled RNA isolated from iBAT and WAT collected from adult B6 mice. Standards and unknown samples were run in duplicate and normalized to cyclophilin B. The average level of cyclophilin B slightly decreased with increasing age from 10 to 63 days of postnatal development; however, it remained stable between 10 and 21D mice raised at different ambient temperatures (17°C *vs.* 29°C) and between 56 and 63D mice reared at 23 or 4°C in each strain. The pattern of expression was the same, regardless of whether the expression of target genes was normalized to cyclophilin B or the RNA input. The 112D mice were analyzed as a separate cohort and were not compared with the other groups. Sequences of probes and primers are available on request.

#### Microarray analysis

Total RNA from the inguinal WAT (ING) of 10 and 21D mice (*n* = 16 per age group) maintained at 17 or 29°C with an RNA integrity number range of 8.6 to 9.4 (2100 Bioanalyzer; Agilent Technologies, Santa Clara, CA, USA) was used for microarray analysis of each mouse. RNA was amplified, labeled, and hybridized onto chips containing more than 56,000 probes (Single Color SurePrint G3 Mouse Gene Expression Microarrays; Agilent Technologies), according to the manufacturer’s protocol. Array images were analyzed with Agilent Feature Extraction software. GeneSpring GX 12 (Agilent Technologies) software was used for absolute and comparative analyses. Quality control after quantile normalization revealed approximately 31,000 probes. Probes below background signal or with sequences unmapped to Ensembl (release 55) transcripts were discarded. The false-discovery rate-corrected *P* value was less than 0.05. Microarray data have been deposited online in the Gene Expression Omnibus repository (National Center for Biotechnology Information, Bethesda, MD, USA), with the accession number GSE62350.

#### Western blot analysis

Adipose tissues were homogenized in RIPA buffer containing protease (PMSF, P7626, and Protease Inhibitor Cocktail P8340; Sigma-Aldrich, St. Louis, MO, USA) and phosphatase inhibitors (Pierce Phosphatase Inhibitor Mini Tablets 88667; Thermo Fisher Scientific) and incubated on ice for 1 hour. After centrifugation (10,000 *g*, 15 minutes, 4°C), the supernatants were collected, and protein concentration was determined with Bradford reagent (B6916; Sigma-Aldrich). Western blot analysis was performed as described by Xue *et al.* (7), with goat anti-uncoupling protein (UCP)-1 (sc-6529; Santa Cruz Biotechnology, Dallas, TX, USA) and mouse anti-β-actin (ab6276; Abcam, Cambridge, United Kingdom). The bands were visualized with an Odyssey imaging system (LI-COR Bioscience, Lincoln, NE, USA) with fluorescence-labeled secondary antibodies (IRDye800 and IRDye700; LI-COR), according to the manufacturer’s protocol. UCP1 signals were normalized to β-actin.

#### Histology

Fat tissues were fixed in 6% formalin, embedded in paraffin, and stained with hematoxylin and eosin for general morphologic examination. Cells expressing UCP1 were identified immunohistochemically, according to the avidin-biotin complex (ABC) method (Vector Laboratories, Burlingame, CA, USA). Endogenous peroxidase activity was blocked with 3% hydrogen peroxide in methanol. Tissue fragments were incubated with 10% normal goat serum for 1 hour followed by overnight incubation with primary antibody against UCP1 (a kind gift of Drs. Pavel Flachs and Jan Kopecky, Department of Adipose Tissue Biology, Institute of Physiology, Academy of Sciences of the Czech Republic, Prague, Czech Republic) at 4°C. The antibody was detected with rabbit anti-goat IgG biotin-conjugated secondary antibody, followed by ABC reagent (Vector Laboratories). The enzymatic reaction to reveal peroxidase was performed with 3,3-diaminobenzidine tetrahydrochloride hydrate (D5637; Sigma-Aldrich). The tissue sections were counterstained with hematoxylin.

#### Food intake

The food intake (FI) of the lactating females was monitored within a 3 day interval between 7 and 10 days of lactation of the newborn. The value was corrected by the FI of the male, estimated at 3 g/d. FI in the adult male mice was measured as shown in Supplemental Fig. S1*D*.

#### Total energy expenditure measurements

Total energy expenditure (TEE) of the pups was measured as described by Ravussin [TEE = metabolizable energy intake (MEI) – (∆somatic fat energy + ∆somatic fat-free energy)] (21). Briefly, the FI of the lactating females was corrected by both the FI of the males and the FI of the nonlactating females, estimated at 3 g/d each. The obtained value was treated as the MEI. The cumulative changes in FM and lean mass (LM) of the 8 pups per litter between 7 and 10 days of age constituted the somatic fat and fat-free energy, respectively. Metabolic efficiency (ME) was estimated as the cumulative changes in FM and LM relative to the estimated FI of the pups. We assigned caloric equivalents for changes in FM = 9.0 kcal/g and LM = 4.0 kcal/g (22) and 3.4 kcal/g for food consumed (PicoLab Rodent Diet 20 5053; LabDiet). The number of litters analyzed was 6 and 11 for the AxB8 mice at 17 and 29°C, and 11 and 9 for the B6 mice at 17 and 29°C, respectively.

#### Glucose tolerance test

The mice were not fed for 4 hours and were weighed before intraperitoneal glucose injections. For the glucose tolerance test (GTT), the glucose dose (20% in saline solution) was calculated for 2 mg/kg BW. Blood was collected by tail snipping. Blood glucose levels were measured at 0, 15, 30, 60, 90, and 120 minutes after injection.

#### Plasma parameters

Plasma insulin and leptin were measured with ELISA kits (Wide Range Mouse Insulin Immunoassay kit; Biorbyt Ltd., Cambridge, United Kingdom; Mouse/Rat Leptin ELISA kit; Phoenix Pharmaceuticals, Inc., Burlingame, CA, USA).

#### CL 316243 and thyroid hormone administration

Mice reared at 29°C received saline (control) or the β3-adrenergic receptor agonist CL 316243 at [1 mg/kg BW (6)] injection daily for 3 consecutive days, starting on postnatal day 7. Additional mice maintained at 17 or 29°C received a control or thyroid hormone [T3; 15 ng/g BW (23), Sigma-Aldrich] by intraperitoneal injection every 24 hours on postnatal days 7, 8, and 9 or 18, 19, and 20. The mice from each litter were subdivided into control and experimental groups and dissected at postnatal day 10 or 21.

### Statistics

Data sets were analyzed with Student’s *t* test or the Mann-Whitney test for single comparisons or with 2-way ANOVA when 2 or more groups were compared. Differences between the means were considered statistically significant if *P* < 0.05. Microarray data were analyzed by GeneSpring GX 12 and Ingenuity Pathway Analysis (IPA) software (Qiagen).

## RESULTS

### Ambient temperature during early development affects energy balance phenotypes

**Figure 1.**
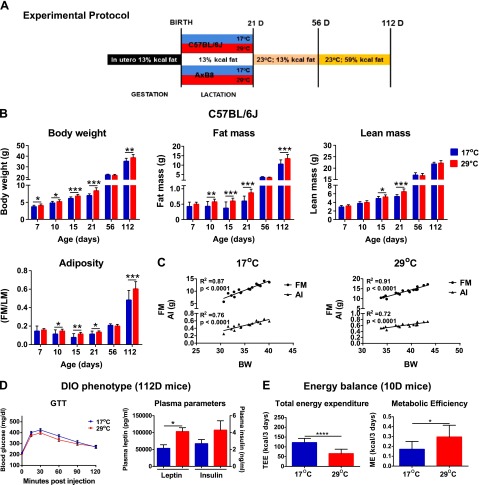
Phenotypic changes in response to variation in ambient temperature during early development. *A*) Experimental protocol. *B*) Adiposity phenotypes of B6 (C57BL/6J) mice (*n =* 16 at 17°C; *n =* 16 at 29°C). *C*) Correlation coefficient analysis between BW and FM and BW and AI (FM/LM), as determined by NMR in 112D B6 mice. *D*) The DIO phenotype determined by GTT and plasma parameters in 112D B6 mice (*n =* 7/group). *E*) TEE and ME in 10D B6 mice (8 pups per litter). Data are expressed as means ± sd. *P < 0.05; **P < 0.01; ***P < 0.001; ****P < 0.0001. See also Supplemental Table S1 and Supplemental Fig. S1.

We predicted that variations in ambient temperature during the early postnatal period would influence body composition in developing mice and would affect susceptibility to DIO in adulthood. We tested this prediction in mice reared at 17 or 29°C from birth to weaning and fed an HFD for 8 weeks from 56 to 112 days of age (**Fig. 1*A***). Figure 1*B* and Supplemental Fig. S1*A* track the body composition of the same mice from birth to 112 days of age. Supplemental Table S1 presents the complete data set including mice killed at intermediate times for tissue collection for molecular analysis. Both data sets show that mice reared at 17°C before weaning had significantly lowered FM, BW, and adiposity index (AI) (Fig. 1*B*, Supplemental Fig. S1*A*, and Supplemental Table S1). The suppression of FM and adiposity by 17°C occurred first between 7 and 21 days of age in both strains of mice. In addition, LM was modestly reduced in both the 21D B6 and AxB8 mice (*P* ≤ 0.0003; Supplemental Table S1, *P* < 0.05). Of note, phenotypic differences in FM and LM content between mice reared at 17 or 29°C during lactation were no longer detectable at 56 days of age after the mice had been maintained for 5 weeks on a low-fat STD at 23°C. However, 112D B6 and AxB8 mice reared at 17°C showed a 22 and 23% reduction in FM accumulation after being fed an HFD, compared with mice raised at 29°C.

In general, the B6 mice had markedly higher BW and LM compared with the AxB8 mice (*P* < 0.05). The adult B6 mice accumulated more fat than did the AxB8 mice when fed an HFD, regardless of the ambient temperature. Total energy intake (in kilocalories per gram per week) remained unaltered in both strains of mice with respect to external environment (Supplemental Fig. S1*D*). Despite a slight decrease in fasting glucose levels in the AxB8 mice (*P* < 0.01), the GTT showed no differences in glucose tolerance (Fig. 1*D* and Supplemental Fig. S1*C*), although insulin and leptin plasma levels were significantly lower in both the B6 and the AxB8 mice reared at 17°C. Two-way ANOVA (Supplemental Table S1) showed that ambient temperature had an exclusive effect on FM before weaning, and it strongly affected the DIO phenotype in adult mice. BW correlated strongly with FM and AI in 112D mice in both strains, independent of ambient temperature (Fig. 1*C* and Supplemental Fig. S1*B*).

Lactating dams from both strains of mice that were acclimated to 17°C consumed considerably more food within a 3 day interval (between days 7 and 10 during lactation) than did the dams raised at 29°C (Supplemental Fig. S1*E*), whereas their BW remained unaltered (Supplemental Fig. S1*F*). This result indicates that, under conditions of increased EE, lactating mice preferentially increase FI, rather than burn their internal energy reserves. Moreover, 17°C significantly stimulated TEE and decreased ME in the 10D B6 (Fig. 1*E*) and AxB8 (Supplemental Fig. S1*G*) mice, estimated by using the modified energy balance method described by Ravussin *et al.* (21). The greater ME obtained by pups reared at 29°C confirmed the increased fat accumulation evidenced by NMR data.

### The expression of BA biomarker genes during early postnatal development

**Figure 2.**
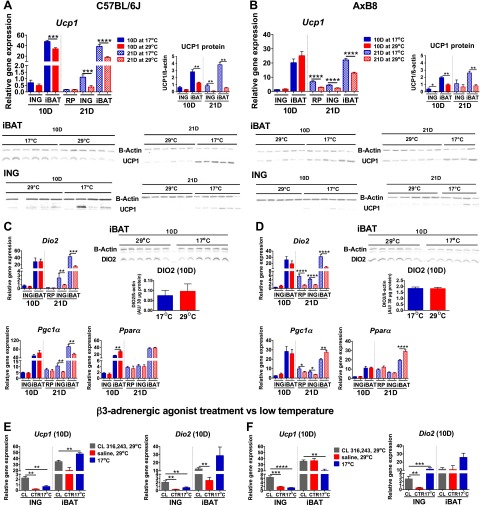
Gene expression associated with brown adipocyte induction and iBAT function during early postnatal development. *A*) *Ucp1* gene and protein expression profiles in WAT and iBAT of 10D (*n =* 8 at 17°C; *n =* 8 at 29°C) and 21D (*n =* 12 at 17°C; *n =* 15 at 29°C) B6 (C57BL/6J) mice. *B*) *Ucp1* gene and protein expression profiles of 10D (*n =* 12 at 17°C; *n =* 14 at 29°C) and 21D (*n =* 14 at 17°C; *n =* 14 at 29°C) AxB8 mice. *Dio2*, *Pgc1a*, and *Ppara* gene expression profiles in 10 and 21D B6 (*C*) and AxB8 mice (*D*)*. Ucp1* and *Dio2* gene expression profiles in 10D B6 (*E*) and AxB8 (*F*) mice injected with CL 316243 (CL) or saline (CTR). Graphs include a comparison between CL-injected mice and pups raised at 17°C. See also Supplemental Fig. S2. Data are expressed as means ± sem. *P < 0.05; **P < 0.01; ***P < 0.001; ****P < 0.0001, for rearing temperature effects only for panels *A*–*D* and for the significance of treatments for panels *E* and *F*.

Variation in peroxisome proliferator-activated receptor α (*Ppara*), peroxisome proliferator-activated receptor γ coactivator 1 α (*Pgc1a*), and type II iodothyronine deiodinase (*Dio2*) mRNA levels correlates highly with differences in *Ucp1* expression in retroperitoneal WAT (RP) between cold-exposed A/J and B6 mice (7). These earlier findings, as well as those of Waldén *et al.* (24), indicate that the former genes are useful biomarkers for BAs and potential differences in the expression of *Ucp1* between B6 and AxB8 mice during cold stimulation. A key finding [**Fig. 2*A*, ***C* (B6) and *B*, *C* (AxB8)] shows that *Ucp1*, *Dio2*, and *Pgc1a* mRNA levels, as well as UCP1 protein in ING from 10D mice were not significantly increased by 17°C. In contrast, the expression of BA biomarker genes was up-regulated in ING from 21D mice reared at 17°C, together with induction of UCP1 protein. Whereas iBAT *Ucp1* mRNA was increased only in 10D B6 mice, iBAT UCP1 protein was elevated in both strains of mice raised at 17°C. At 21 days of age, iBAT *Ucp1* mRNA and UCP1 protein levels were up-regulated in both the B6 and the AxB8 mice reared at 17°C, compared with those raised at 29°C.

Although, there were no temperature-dependent differences in the expression of brown fat biomarker genes in ING from 10D mice, intraperitoneal injections of CL 316243 for 3 consecutive days, starting on day 7 of postnatal development, up-regulated the expression of *Ucp1* and *Dio2* in ING from the 10D B6 (Fig. 2*E*) and AxB8 (Fig. 2*F*) mice reared at 29°C. Notably, CL 316243 was about 4 times more effective in inducing *Ucp1* than 17°C. In addition, *Dio2* mRNA levels were increased in iBAT from the 10D B6 mice, whereas the expression of *Pgc1a* and *Ppara* in both ING and iBAT remained unaffected by administration of a β3-adrenergic receptor agonist (Supplemental Fig. S2*A, B*) and resembled the expression profiles observed in the mice raised at 17 or 29°C.

Similarities in gene expression patterns and a strong correlation between *Ucp1* and *Dio2* mRNA levels suggested that T3 signaling regulates early postnatal BA development. The expression of thyroid hormone receptor β (*ThRB*) was increased by 8.1- and 1.5-fold in ING from the 10D B6 and AxB8 mice maintained at 17°C compared to those raised in 29°C, whereas at 21 days of age, changes in the *ThrB* mRNA levels were evident only in the B6 mice (Supplemental Fig. S2*C,D*). Accordingly, we sought to augment a possible deficiency in T3 production caused by low DIO2 levels by intraperitoneal T3 administration. Similar to CL 316243 treatment, we conducted a series of daily T3 injections within a 3 day period, starting when the pups were either 7 or 18 days of age and dissected the mice at postnatal days 10 and 21. Our data showed no differences in the expression of brown fat biomarker genes in the T3- or saline-injected 10D mice (Supplemental Fig. S2*E, F*). T3 injections from postnatal day 18 to 20 resulted in an ∼3-fold increase in *Ucp1* mRNA in ING from the 21D B6 and AxB8 mice raised at 29°C, whereas there was virtually no effect in the 21D mice maintained at 17°C (Supplemental Fig. S2*G, H*).

### Postnatal development of BAs in WAT: global microarray analysis of ING

**Figure 3.**
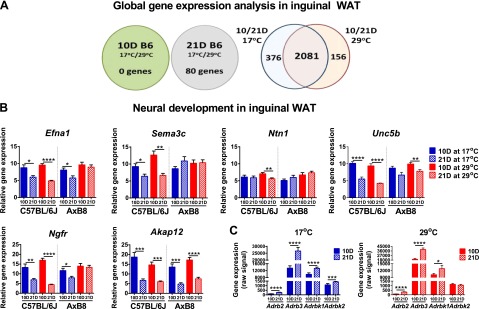
Global gene expression analysis in ING. A) Diagrams showing the results of microarray analyses of 10 and 21D B6 (C57BL/6J) mice, presenting the number of differentially expressed genes with an FC (abs) > 1.5*. B*) Expression of genes of neural development in the developing B6 and AxB8 mice*. C*) Expression of β3-adrenergic signaling genes determined by microarray analyses in 10 and 21D B6 mice. Data are expressed as means ± sem. *P < 0.05; **P < 0.01; ***P < 0.001; ****P < 0.0001. See Supplemental Table S2.

The lack of differences in the expression of BAT biomarker genes between the 10D mice reared at 17 or 29°C (Fig. 2*A, B*) suggested that ING from the 10D mice did not respond to sympathetic stimulation by 17°C. However, the experiment with CL 316243 (Fig. 2*E, F*) indicated that ING responded to treatment with a β3-adrenergic receptor agonist. To confirm this observation, we performed a high-density microarray analysis, to compare global gene expression between ING from 10D B6 mice maintained at 17 or 29°C. We detected more than 31,600 transcripts, of which only 3 were expressed at significantly different levels with respect to ambient temperature [*Ptprb*, protein tyrosine phosphatase receptor type B, fold change (FC) = 1.40; *Cog5*, component of oligomeric Golgi complex 5 (FC = 1.33); and *Zfp59*, zinc finger protein 59 (FC = 1.26); *P* (corr) < 0.05]. This finding indicates that the regulation of gene expression in ING up to 10 days of age was not affected by the 17°C ambient temperature that should have stimulated sympathetic activity. Seeing that brown fat biomarkers were induced by 17°C at 21 days of age (Fig. 2*A*, *C*), we analyzed global expression in ING from 21D B6 mice. In contrast to the 10D pups, in the 21D mice, 17°C modulated the expression of 80 genes (FC > 1.5; **Fig. 3*A***), of 30,702 targets, that are associated with carbohydrate and lipid metabolism as well as mitochondrial function [*Cyp2b10*, cytochrome P450, family 2, subfamily *b*, polypeptide 10; *Ucp1*; *Cyp2f2*, cytochrome P450, family 2, subfamily *f*, polypeptide 2; *Gyk*, glycerol kinase; *Acot11*, acyl-CoA thioesterase 11; *Cox7a1*, cytochrome *c* oxidase subunit VIIa polypeptide 1 (muscle); *Gpd2*, glycerol phosphatase dehydrogenase 2, mitochondrial; *Cox8b*, cytochrome *c* oxidase subunit VIIIb; and *Cidea*, cell death-inducing DFFA-like effector a; Supplemental Table S2]. These results suggest that ING from 21D mice responds to cold stimulation, a trait undeveloped in 10D mice.

To identify molecular changes and signaling pathways that influence the development of sympathetic activity in ING in response to 17°C, we compared gene expression between the 10 and 21D B6 mice (Fig. 3*A*). Venn diagram analysis revealed that of 2457 and 2237 genes differentially expressed with age at 17 and 29°C, respectively, 2081 genes were differentially expressed at both temperatures, whereas the expression of an additional 376 was uniquely different at 17°C, and that of another 156 genes was uniquely different at 29°C. Using IPA, we determined that genes differentially expressed with age, irrespective of the ambient temperature, constituted transcripts involved in the formation and growth of plasma membrane projections, cytoskeleton organization, and cell spreading (**Table 1**), as well as protein kinase A and phosphatase and tensin homolog deleted from chromosome-10 (PTEN) signaling pathways (**Table 2**). In addition, we detected genes of axonal guidance, ephrin A signaling, neuritogenesis, and dendritic cell maturation, suggestive of the development of neural structures in ING (Tables 1 and 2). These results demonstrate that acquisition of the BAP in ING is functionally linked to axon growth and guidance (*P* = 1.28E-11) between 10 and 21 days of age, further evidenced by age-dependent differences in the expression of numerous genes of neural signaling (**Table 3**). Validation of microarray results using quantitative RT-PCR revealed that the expression of nerve growth factor receptor (*Ngfr*), ephrin-a1 (*Efna1*), A-kinase anchor protein 12 (*Akap12*), semaphorin 3c (*Sema3c*), netrin-1 (*Ntn1*), and transmembrane receptor Unc-5 homolog B (*Unc5b*) were increased in the ING of the 10D mice of both strains (Fig. 3*B*). In support of evidence for the formation of sympathetic responsiveness in ING, adrenergic β receptor kinase 1 (*Adrbk1*) and 2 (*Adrbk2*) as well as adrenergic receptor β2 (*Adrb2*) and β3 (*Adrb3*) mRNA levels were increased between 10 and 21 days of age (Fig. 3*C*).

**TABLE 1:** Functions regulated between 10 and 21 days of age in ING from B6 mice

Category/function	*P*	Activation *z*-score	Molecules (*n*)
Cell morphology			
Cell spreading	4.88E-16	2.577	77
Sprouting	9.83E-11	2.787	78
Cellular assembly and organization			
Formation of plasma membrane projections	6.86E-12	2.593	110
Growth of plasma membrane projections	6.72E-11	3.268	101
Outgrowth of plasma membrane projections	8.23E-11	3.638	91
Formation of cellular protrusions	4.25E-21	3.509	172
Organization of cytoskeleton	2.87E-28	3.986	268
Organization of cytoplasm	1.33E-25	4.042	276
Microtubule dynamics	1.49E-21	3.601	221
Cellular movement			
Migration of endothelial cells	1.08E-10	2.030	73
Migration of cells	3.05E-41	2.393	407
Cell movement of fibroblasts	9.16E-08	2.458	42
Cell movement of neuroglia	6.00E-07	2.484	26
Cell movement	1.82E-41	2.554	440
Nervous system development and function			
Axonogenesis	2.95E-07	3.289	41
Neuritogenesis	7.78E-13	2.377	104
Morphogenesis of neurites	1.68E-07	2.403	67
Growth of neurites	1.01E-10	3.395	100
Outgrowth of neurites	1.55E-10	3.765	90
Growth of nervous tissue	6.35E-13	4.097	125

Genes included in the analyses had an absolute FC > 1.5. The predicted activation state observed at 10 days was “increased” for all categories and functions.

**TABLE 2.** Signaling pathways regulated between 10 and 21 days of age in ING from B6 mice

Ingenuity canonical pathway	*P*	Down-regulated genes (10D/21D)	Up-regulated genes (10D/21D)	Ratio
Axonal guidance	1.28E-11	15/487	67/487	82/487
Integrin signaling	2.85E-05	9/208	26/208	35/208
Protein kinase A signaling	3.61E-05	24/409	34/409	58/409
Notch signaling	5.07E-05	1/43	11/43	12/43
Phospholipase C signaling	2.05E-04	24/265	14/265	38/265
Reelin signaling in neurons	3.26E-04	8/85	9/85	17/85
Paxillin signaling	3.29E-04	6/117	14/117	20/117
Actin cytoskeleton signaling	4.88E-04	10/242	24/242	34/242
Gap junction signaling	5.62E-04	9/181	19/181	28/181
Epithelial adherens junction signaling	1.81E-03	4/154	20154	24/154
Dendritic cell maturation	2.65E-03	20/211	7/211	27/211
Netrin signaling	3.51E-03	2/58	8/58	10/58
PTEN signaling	1.40E-02	8/139	10/139	18/139
Ephrin A signaling	2.51E-02	3/54	6/54	9/54

**TABLE 3.** Genes of axonal guidance differentially expressed with age in ING at both ambient temperatures

Gene symbol	Gene name	Fold change (10D/21D)	*P* (corr)	Raw signal 10D	Raw signal 21D
*Ablim3*	Actin binding LIM protein family, member 3	2.19	9.65 E-7	12851.34	5096.22
*Akap12*	A kinase (PRKA) anchor protein 12	2.55	3.63E-6	23995.28	7418.80
*Bmp1*	Bone morphogenetic protein 1	2.07	3.15E-4	3347.90	1376.44
*Bmp3*	Bone morphogenetic protein 3	4.69	5.47E-7	1556.17	277.67
*Cxcr4*	Chemokine (C-X-C motif) receptor 4	−2.56	4.36E-4	345.48	736.95
*Efna1*	Ephrin A1	2.11	2.61E-6	3147.43	1260.76
*Epha2*	Eph receptor A2	1.55	1.30E-3	962.28	519.21
*Epha3*	Eph receptor A3	2.48	7.19E-6	423.09	142.28
*Ephb4*	Eph receptor B4	1.58	1.78E-5	1823.39	1154.75
*Fzd1*	Frizzled family receptor 1	1.61	1.57E-4	3294.49	1737.36
*Fzd2*	Frizzled family receptor 2	2.30	5.65E-5	1510.22	553.03
*Fzd4*	Frizzled family receptor 4	2.13	1.12E-6	27910.91	11573.53
*Gli1*	GLI family zinc finger 1	3.69	6.92E-7	1546.43	352.42
*Gli3*	GLI family zinc finger 3	1.88	7.20E-6	875.61	391.09
*Glis2*	GLIS family zinc finger 2	2.40	2.61E-6	5800.04	2071.55
*Neo1*	Neogenin 1	1.64	6.59E-4	1993.71	1025.67
*Ngfr*	Nerve growth factor receptor	3.04	1.78E-7	1908.67	529.36
*Ntn1*	Netrin1	1.93	2.53E-4	847.38	369.33
*Nrp1*	Neuropilin 1	1.78	2.74E-5	2056.64	974.00
*Nrp2*	Neuropilin 2	1.76	8.48E-7	12442.03	6130.56
*Ntrk3*	Neurotrophic tyrosine kinase receptor, type 3	1.62	3.10E-3	2443.41	1275.19
*Plxna3*	Plexin A3	1.83	8.56E-4	342.15	156.10
*Plxna4*	Plexin A4	1.83	2.01E-5	665.49	303.65
*Plxnb3*	Plexin B3	1.83	1.69E-4	392.32	178.84
*Plxnc1*	Plexin C1	−1.53	2.22E-2	655.39	836.41
*Plxnd1*	Plexin D1	1.51	2.69E-5	1811.96	1006.60
*Sema3c*	Semaphorin 3C	2.64	9.06E-4	9006.91	2944.58
*Sema4d*	Semaphorin 4D	−1.73	1.94E-2	1183.27	1717.65
*Sema5b*	Semaphorin 5B	5.52	2.70E-7	300.19	44.94
*Slitrk6*	Slit and Ntrk-like family, member 6	5.01	1.53E-6	303.93	50.48
*Smo*	Smoothened, frizzled family receptor	1.73	1.69E-6	11376.56	5678.09
*Srgap1*	Slit-Robo Rho GTPase activating protein 1	2.03	1.10E-3	239.78	98.54
*Tuba1a*	Tubulin, α 1a	2.98	1.01E-6	86573.28	27387.47
*Tuba8*	Tubulin, α 8	−2.59	2.26E-3	1037.29	2270.74
*Unc5a*	Unc-5 homolog A (*C. elegans*)	1.87	2.10E-3	4363.89	1992.32
*Unc5b*	Unc-5 homolog B (*C. elegans*)	2.71	4.10E-8	3761.43	1171.69

The expression of the chosen genes with an absolute FC > 1.5 (*n =* 8 mice/group) is shown. Fold change difference was generated based on normalized signals obtained from mice reared at 29°C (data not shown).

In addition to temperature-independent changes in ING between 10 and 21 days of age, the expression of genes of mitochondrial function and the BAP were up-regulated at 17°C [*e.g.*, *Cyp2b10* (FC = 251.69); *Acot11* (FC = 3.48); *Cidea* (FC = 3.48); *Cpt1b*, carnitine palmitoyltransferase 1B (muscle) (FC = 2.94); *Cox8b* (FC = 2.42); *Dio2* (FC = 2.38); and *Cox7a1* (FC = 2.35)], indicative of the maturation of the BAP between 10 and 21 days of age. Except for *Cyp2b10* (FC = 20.37), none of these changes was among the 156 transcripts found in the animals raised at 29°C.

### Involution and reemergence of the BAP during cold exposure

**Figure 4.**
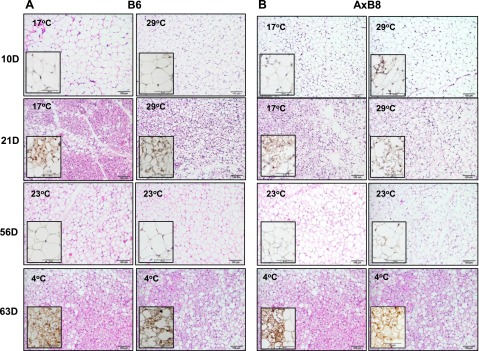
Histologic examination of inguinal BAP**.** Sections of ING from 10, 21, 56 (moved to 23°C at weaning), and 63D (moved to 23°C at weaning and exposed to 4°C for 7 days) B6 (C57BL/6J) (*A*) and AxB8 (*B*) mice maintained at 17 or 29°C during the preweaning period. Large images show the complex patterns of reversible white and brown adipocyte morphology as a function of age and ambient temperature, stained with hematoxylin and eosin. Scale bar, 100 µm. Inset images show anti-UCP1 immunohistochemical staining, counterstained with hematoxylin. Scale bar, 50 µm.

**Figure 5.**
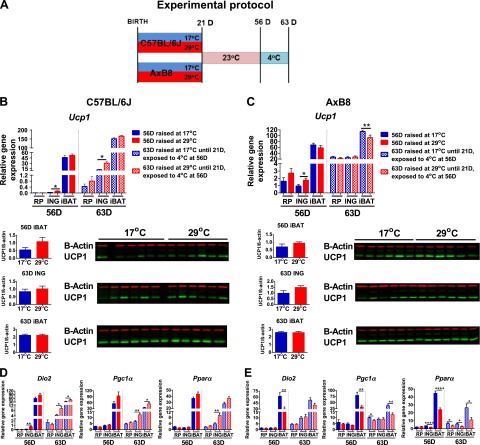
Reversible expression of BAT biomarker genes in RP, ING, and interscapular fat from adult mice maintained at 23°C or exposed to 4°C for 7 days**.** *A*) Experimental protocol. Expression profiles of *Ucp1* mRNA and protein levels in 56 and 63D B6 (C57BL/6J) (*B*) and AxB8 (*C*) mice. Corresponding mRNA levels of *Dio2*, *Pgc1a*, and *Ppara* in 56 and 63D B6 (*D*) and AxB8 (*E*) mice (*n =* 7–10 mice/group). Data are expressed as means ± sem. *P < 0.05; **P < 0.01; ***P < 0.001; ****P < 0.0001. Significances are for the effects of rearing temperature.

**Figure 6.**
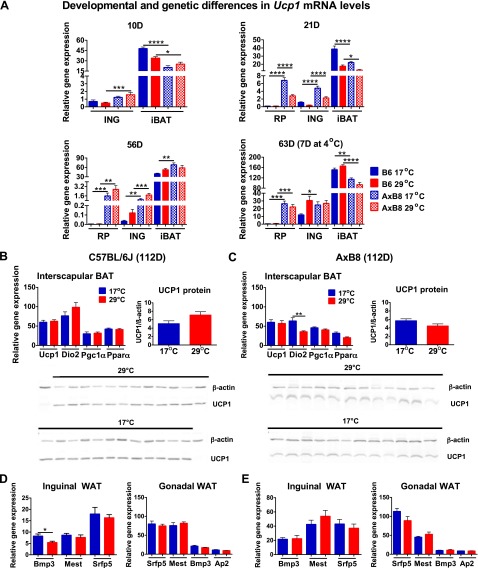
Genetic and developmental differences between the interscapular and WAT brown adipogenesis programs**.** *A*) Developmental and genetic differences in *Ucp1* mRNA levels between B6 (C57BL/6J) and AxB8 mice. Interscapular BAT gene and protein levels in 112D B6 (*B*) and AxB8 (*C*) mice (*n =* 11–16). The expression of WAT expansion genes in ING and epididymal WAT of adult B6 (*D*) and AxB8 (*E*) mice. Data are expressed as means ± sem. *P < 0.05, *P* < 0.01, *P* < 0.001, *P* < 0.0001.

ING collected from the 10D B6 (**Fig. 4*A***) and AxB8 (Fig. 4*B*) mice, reared at 17 or 29°C, resembled typical unilocular WAT with virtually no multilocular cells characteristic of BAs. A few clusters of cells positive for UCP1 were detected in the AxB8 mice reared at 17°C, but not in the B6 mice. Histologic sections of ING from the 21D B6 and AxB8 mice were dominated by BA-like cells, irrespective of the ambient temperature. Sections of ING from 21D mice reared at 29°C showed large regions of brown-like adipocytes with enlarged lipid vacuoles, resembling iBAT from mice reared at thermoneutrality or deficient in UCP1 (25). Whereas ING sections from 10D mice were UCP1 negative (Fig. 4, inset images) at both 17 and 29°C, UCP1-positive signals were obtained for multilocular cells dispersed within ING from 21D mice reared at both ambient temperatures, albeit with a more intense UCP1-positive reaction found for mice raised at 17°C. These results, together with BAT gene expression data, indicate that ING undergoes its developmental program, which includes assembling BAs at 21 days of age, irrespective of external environment. Next, we tested the idea that if the number of BAs is determined genetically between birth and weaning, then reemergence of the BAP in WAT of adult mice exposed to cold would be similar, irrespective of prior rearing conditions (**Fig. 5*A***). Although the BAP was present in the 21D mice, after 5 weeks at 23°C, ING resembled traditional WAT with no trace of BAs or UCP1-positive signals clearly visible in the mice at weaning (Fig. 4*A, B*). In addition, the expression of BAT biomarker genes in WAT from 56D mice was very low, despite prior ambient temperature, with undetectable levels of UCP1 protein (Fig. 5*B, C*). After 7 days at 4°C, the ING acquired a brown fat morphology (Fig. 4*A, B*) that was indistinguishable in the 63D B6 (Fig. 5*B, D*) and AxB8 (Fig. 5*C*, *E*) mice, despite the previous environmental conditions. Exposure to 4°C robustly induced the expression of thermogenic genes (∼300-fold for *Ucp1* in B6 and ∼25-fold in the AxB8) in mice maintained at both 17 and 29°C before weaning. The sole major significant difference was the higher levels of BAT biomarker genes in the RP of the AxB8 mice, compared with levels in the B6 mice (**Fig. 6*A***). Despite a similar response to ambient temperature in both strains of mice, in general, *Ucp1* expression in ING was more robust in the AxB8 mice. These results indicate that the thermogenic potential of brite cells from B6 mice remains equal, irrespective of environmental conditions from birth to weaning. Five weeks after weaning, the expression of thermogenic genes present at 21 days of age regressed, but they were reinduced to similar levels in the mice reared at 17 or 29°C when the temperature was reduced to 4°C. The same applies to the AxB8 mice, except that these mice have a higher capacity for BA induction determined from a genetically variable developmental program (26) that may be based on the number of brite cells, as suggested by immunohistology of retroperitoneal fat from recombinant inbred strains with variable *Ucp1* induction (6).

### Adipose tissue phenotypes in adult mice with DIO

Despite a slight increase in *Dio2* in the AxB8 mice, 17°C during early postnatal development did not affect the expression of iBAT biomarker genes in the 112D mice from both strains (Fig. 6*B, C*). Moreover, the amount of UCP1 protein in iBAT from those mice was indistinguishable with respect to ambient temperature. Physiologic data suggest that WAT expansion is greater in the 112D mice fed an HFD for 8 weeks and maintained at 29°C during the preweaning period. Therefore, we measured the expression of genes associated with WAT expansion (*Bmp3*, bone morphogenetic protein 3; *Mest*, mesoderm-specific transcript, and *Sfrp5,* secreted frizzled*-*related protein 5) (27–29). The analyses of inguinal and epididymal WAT revealed no temperature-dependent differences in the expression of WAT biomarker genes in either strain of mice (Fig. 6*D, E*).

## DISCUSSION

This study on the effects of ambient temperature during the early postnatal period revealed novel features of BA development in white fat. First, sympathetic activity in WAT in response to low ambient temperature was not present until the mice were between 10 and 21 days of age. Second, the BAP was more intensely expressed in 21D mice reared at 17°C, but this difference was lost when the mice were subsequently maintained at the same temperature—that is, at 23°C from 21 to 56 days of age and at 4°C from 56 to 63 days. This result suggests that the thermogenic potential of brite cells is determined genetically between birth and weaning. Third, the involution of the BAP in WAT occurred between 21 and 56 days of age. Recently, an investigation of the development of brown fat in the lamb described a perirenal tissue that has the cellularity and precocious expression of UCP1 at birth, similar to mouse iBAT, but loses the brown phenotype within 7 days after birth, somewhat similar to brite cells in the mouse (30).

Molecular and morphologic data showed that ING from 10D pups did not respond to SNS stimulation at an ambient temperature of 17°C, despite a significant reduction in FM, indicative of the increased fatty acid (FA) oxidation that supplies substrates for the activation of classic iBAT thermogenesis. The preweaning *Ucp1^−/−^* mice do not survive an ambient temperature of 23°C, and therefore, an active UCP1-dependent thermogenesis is essential in the neonatal mouse (31). As FAs are abundant in mouse milk (32) newborn pups may use those ingested directly from mother’s milk. A greater energy content of milk produced by dams kept at 8°C (33) suggests higher availability of FAs for pups raised at 17°C. Excess FAs supplied with milk may be deposited in WAT, resulting in increased adiposity at 29°C. We observed no temperature-dependent differences in the expression of adipose triglyceride lipase (*Pnpla2*; patatin-like phospholipase domain-containing protein 2); hormone-sensitive lipase (*Lipe,* mouse hormone-sensitive lipase); and other lipolytic enzymes [*e.g.*, *Mgll,* monoacylglycerol lipase, or *Lpl,* lipoprotein lipase (data not shown)]. *Pnpla2* mRNA levels were slightly increased with age, whereas *Mgll* and *Lpl* levels were higher in the 10D mice, suggesting that the FAs for iBAT thermogenesis during early development could be supplied by lipases, independent of β-adrenergic stimulation.

Although 17°C did not stimulate the expression of BAT biomarker genes in 10D mice (Fig. 2*A, B*), administration of CL 316243 increased *Ucp1* and *Dio2* mRNA levels in ING from the 10D B6 (Fig. 2*E*) and AxB8 (Fig. 2*F*) mice reared at 29°C. Since both cold and CL 316243 induce BA in WAT of adult mice (5–7), induction of *Ucp1* in response to CL 316243, but not by an ambient temperature of 17°C, at 10 days, may be caused by the inability of the CNS to properly integrate and transmit the ambient temperature signals to WAT in the neonatal mouse. The hypothalamic-pituitary-adrenal axis in the mouse is activated at around day 12 of postnatal development (34), and it is possible that the central thermoregulatory network controlling BAT thermogenesis in WAT becomes functional after 10 days of age. The inability to respond to cold stimulation at 10 days of age may result from immature neural innervation and an improper signal transduction cascade from the CNS to the WAT; however, at this time, the development of the central regulatory neural circuits for cold-activated thermogenesis is unknown. The comparative microarray analysis of gene expression in ING revealed that major development of WAT occurred between 10 and 21 days and that much of it was independent of the ambient temperature (Fig. 3*A*). The modulation of expression of genes involved in neural signaling [*e.g.*, *Akap12* (35), *Efna1* (36), *Ntn1*, and *Unc5b* (37)] occurred between 10 and 21 days of age, independent of the ambient temperature. Therefore, the ability to respond to cold stimulation and subsequent acquisition of the BAP may rely on WAT remodeling during the early postnatal period. An ambient temperature of 17°C activated thermogenesis in 21D mice when the capacity to respond to the SNS stimulation by cold had developed. In contrast, the expression of brown fat biomarker genes was markedly lower in WAT from the 21D mice reared at 29°C, indicating an attenuated induction of the BAP.

The question of the origin of brite cells in adult mice is controversial. Several groups have found that BAs in adult WAT arise from *de novo* proliferation of progenitors distinct from classic white/BA precursors (3, 38–40). *In vivo* lineage-tracing studies have shown that inducible BAs in subcutaneous WAT differentiate from a subpopulation of stromal cells capable of becoming brown or white adipocytes, depending on external signals (41). Others believe that BAs in WAT appear as a result of white-to-brown transdifferentiation (42). Rosenwald *et al.* (43) reported that a brite and white adipocyte interconversion constitutes a physiologic mechanism of adaptation to cold stimulation in adult mice. Recent gene-tracing studies revealed that BAs in ING, upon cold exposure, arise from preexisting unilocular adipocytes, not as a result of proliferation and recruitment of progenitor cells (44). Our results support a model in which cold activates the BAP in existing adipocytes, whereas the thermogenic capacity of BA in WAT is determined genetically during early development. At 56 days of age, a complete involution of the BAP induced in WAT at weaning was observed (Fig. 4), with no measurable differences in the expression of brown fat biomarker genes between mice raised at 17 or 29°C (Fig. 5). Disappearance of the BAP occurred between 21 and 56 days of age at 23°C, even though this temperature has been said to cause cold stress in mice (45). Upon exposure to 4°C, the BAP was induced in a manner consistent with equal capacity for thermogenesis, irrespective of environmental conditions from birth to weaning. Recently, Contreras *et al.* (46) concluded that BA induction in ING from adult mice requires continuous sympathetic stimulation. The environment, ambient temperature, nutrition (11), or drugs (20) only transiently modulated activation of the BAP, whereas the initial development of brite cells at 21 days of age was independent of environmental factors.

The AxB8 mice were characterized by higher induction of BAs in WAT, with greater differences found in RP than in ING, compared to induction in the B6 mice and consistent with the results of Guerra *et al.* (6). Genetic variation in mice, determined by *Ucp1* mRNA levels indicating greater thermogenic capacity, was evident from 10 days of age at both ambient temperatures (Fig. 6*A*). These differences in brite cell development are in agreement with original studies from our laboratory and a recent study by Lasar *et al.* (47). During cold exposure, differential expression of *Ucp1* and the number of BAs present in the RP was genetically controlled, whereas the genetic differences were not evident in ING (6, 26). Depot-specific variation in the ability to respond to cold stimulation may result from distinct developmental origins for visceral and subcutaneous WAT (48).

Prolonged cold exposure induces hyperplasia of classic BAs in rodents (49). Lee *et al.* (44) showed that cold-stimulated proliferation and differentiation of BA progenitors in iBAT is restricted to a narrow dorsal region of iBAT. In our study, iBAT *Ucp1* mRNA levels were relatively stable in the B6 and AxB8 mice from 10 to 56 days of age, increased in response to 4°C at 63 days, and reverted to basal levels in the 112D mice. This result is consistent with stable *Ucp1* mRNA levels during development in B6 and A/J mice and an induction of *Ucp1* after cold stimulation (2). Purported changes in the number of iBAT cells may reflect cell turnover processes similar to those observed during tissue renewal and regeneration (50). Thus, the net number of BAs may not change, although cold may accelerate cell turnover.

Adiposity was reduced in the developing pups and adult mice with DIO that were reared at 17°C during the lactation period. Behavioral studies have shown that lower ambient temperatures initiate thermoregulatory huddling, a mechanism by which grouped mice reduce heat loss and increase their ME in response to thermal challenge (51, 52). TEE was increased in 10D mice raised at 17°C compared with that in those raised at 29°C, with no accompanying changes in inguinal BAP, although iBAT UCP1 protein was elevated in both the B6 and AxB8 mice kept at 17°C. These results were due to the instability of the mitochondria and UCP1 at 29°C (53). The lack of thermogenesis at 29°C may increase degradation of ubiquitinated UCP1 and mitochondria (54). Despite differences in FM and adiposity between 112D mice fed an HFD for 8 weeks kept at 17°C and those kept at 29°C during the lactation period, no molecular changes in WAT were found. Since there were no temperature-dependent differences in the amount of iBAT UCP1, we are skeptical as to whether increased iBAT thermogenesis during early postnatal development at 17°C is the underlying mechanism for decreased fat accumulation in adult mice. The same differences in DIO were observed in mice undernourished from birth to weaning, suggesting that a critical factor may be the levels of adiposity during early development and not differences in BA induction (11). Reduced adiposity during adulthood may be caused by permanent changes in metabolic rate during the preweaning period. Differences in ME observed between 10D mice raised at 17°C *vs.* 29°C may define their body composition in adult life, consistent with previous studies on malnutrition showing that the early environment has long-term consequences for BW gain (12, 55).

BA development in WAT occurred during the early postnatal period, independent of the external environment. Stimulation of the SNS by cold, a β3-adrenergic agonist, or by diet, only modulated the BAP, whereas the number and thermogenic potential of BAs were determined by a genetic developmental program. Studies using dopamine β-hydroxylase knock-out (DBH KO) mice have indicated that noradrenaline is essential for the regulation of BAT function rather than the number of BAs (56). In addition, DBH KO mice have shown that adrenergic signaling is not required for the postnatal development of the CNS adrenergic system (57). Our results show that the response to cold stimulation and subsequent activation of the BAP in WAT developed between 10 and 21 days of age, in parallel with the acquisition of SNS responsiveness. In the next step, a mechanism for involution of BAs occurred that then worked in concert with reactivation of the BAP on sympathetic stimulation in adult life. Involution of the BAP represents a fundamental difference between BAs in white and interscapular brown fat. In the former, the *Ucp1* phenotype is completely lost during the transition from cold to warmth, whereas iBAT Ucp1 maintains a functional reservoir of thermogenesis even at thermoneutrality. Therefore, a major source of variation in brown adipogenesis is the number of BAs that are allelically variant among inbred strains of mice. Decreased ambient temperature induces the BAP and increases EE in humans (58), and exploiting individual genetic variability in BA induction could therefore lead to devising strategies to maximize the BAP in response to cold or adrenergic agonist stimulation. Given the probable limitations of various pharmacological strategies that increase the amount and activity of BA in humans (59), further understanding of mechanisms regulating the initial development of BAs in WAT could enable new therapeutic interventions to stimulate and retain brown fat as an antiobesity therapy.

## Supplementary Material

Supplemental Data

## Abbreviations

ABC: avidin-biotin complex
*Acot11*: acyl-CoA thioesterase 11
AI: adiposity index
BA: brown adipocyte
*Akap12*: A-kinase anchor protein 12
BAP: brown adipocyte phenotype
BAT: brown adipose tissue
BW: body weight
*Cidea*: cell death-inducing DFFA-like effector a
*Cox7a1*: cytochrome *c* oxidase subunit VIIa polypeptide 1 (muscle)
*Cox8b*: cytochrome *c* oxidase subunit VIIIb
*Cyp2b10*: cytochrome P450, family 2, subfamily *b*, polypeptide 10
D: day-old
DBH KO: dopamine β-hydroxylase knockout
DIO: diet-induced obesity
*Dio2*: iodothyronine deiodinase-2
EE: energy expenditure
*Efna1*: ephrin-a1
FA: fatty acid
FC: fold change
FI: food intake
FM: fat mass
GTT: glucose tolerance test
HFD: high-fat diet
iBAT: interscapular BAT
ING: inguinal white adipose tissue
IPA: Ingenuity Pathway Analysis
*Lpl*: lipoprotein lipase
LM: lean mass
ME: metabolic efficiency
MEI: metabolizable energy intake
*Mgll*: monoacylglycerol lipase
*Ntn1*: netrin-1
*Pgc1a*: peroxisome proliferator-activated receptor γ coactivator 1 α
*Pnpla2*: patatin-like phospholipase domain-containing protein 2
*Ppara*: peroxisome proliferator-activated receptor α
PTEN: phosphatase and tensin homologue deleted from chromosome-10
RP: retroperitoneal white adipose tissue
SNS: sympathetic nervous system
STD: standard chow diet
T3: thyroid hormone, TEE, total energy expenditure
*ThRB*: thyroid hormone receptor β
UCP1: uncoupling protein1
*Unc5b*: transmembrane receptor Unc-5 homolog B
WAT: white adipose tissue
