# Supplementary material for: Low ambient temperature during early postnatal development fails to cause a permanent induction of brown adipocytes
Source: FASEB J. 2015 Apr 20;29(8):3238–52. doi: 10.1096/fj.15-271395 (PMC4511198; doi:10.1096/fj.15-271395)
Supplement: Supplemental Data [file supp_29_8_3238__index.html]

Low ambient temperature during early postnatal development fails to cause a permanent induction of brown adipocytes — Supplemental Data 

# Low ambient temperature during early postnatal development fails to cause a permanent induction of brown adipocytes

## Supplemental Data

**Files in this Data Supplement:**

- Supplemental Data
- Supplemental Data
- Supplemental Data
- Supplemental Data
