# Supplementary material for: Low ambient temperature during early postnatal development fails to cause a permanent induction of brown adipocytes
Source: FASEB J. 2015 Apr 20;29(8):3238–52. doi: 10.1096/fj.15-271395 (PMC4511198; doi:10.1096/fj.15-271395)
Supplement: Supplemental Data [file supp_fj.15-271395_Supplemental_Figure1.pdf]

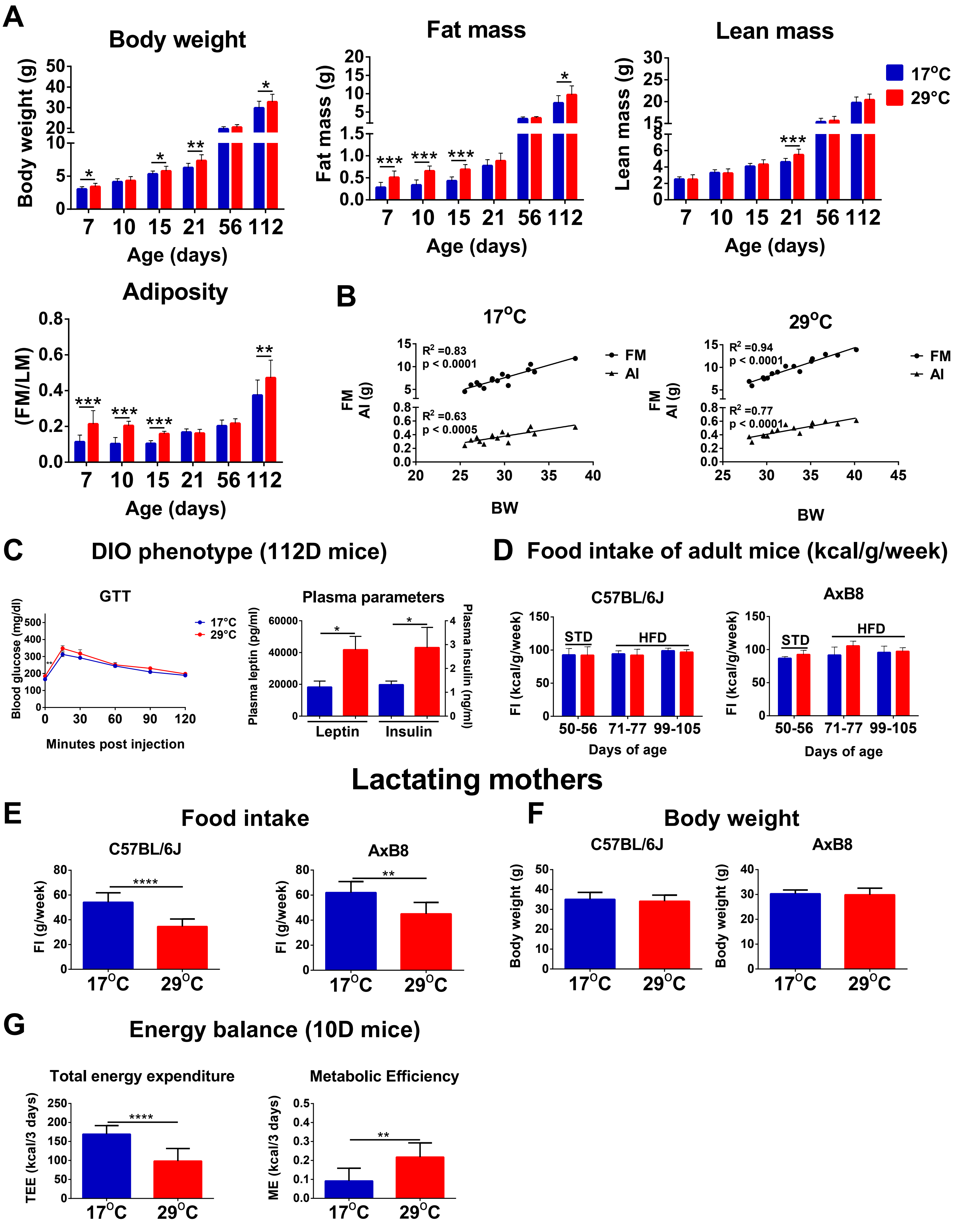

Figure S1. The phenotypic characterization of mice (related to Figure 1). A) Adiposity phenotypes of AxB8 mice (n=15 at 17°C, n=15 at 29°C). B) Correlation coefficient analysis between BW and FM and BW and adiposity index (FM/LM) as determined by NMR in 112D AxB8 mice. C) Diet-induced obesity phenotype determined by glucose tolerance test (GTT) and plasma parameters in 112D AxB8 mice (n=7/group). D) Weekly food intake of adult B6 and AxB8 mice presented as a value of kcal/g/week. E) Food intake (g/week) and (F) body weight of lactating B6 (n= 17 at 17 °C, n=13 at 29 °C) and AxB8 females (n=8 at 17 °C, n=12 at 29 °C). G) Total energy expenditure and metabolic efficiency in 10D AxB8 mice (8 pups/ litter). Data are mean ± SD. * $p<0.05$; ** $p<0.01$; *** $p<0.001$.
