# Supplementary figures and images for: Low ambient temperature during early postnatal development fails to cause a permanent induction of brown adipocytes
Source: FASEB J. 2015 Apr 20;29(8):3238–52. doi: 10.1096/fj.15-271395 (PMC4511198; doi:10.1096/fj.15-271395)

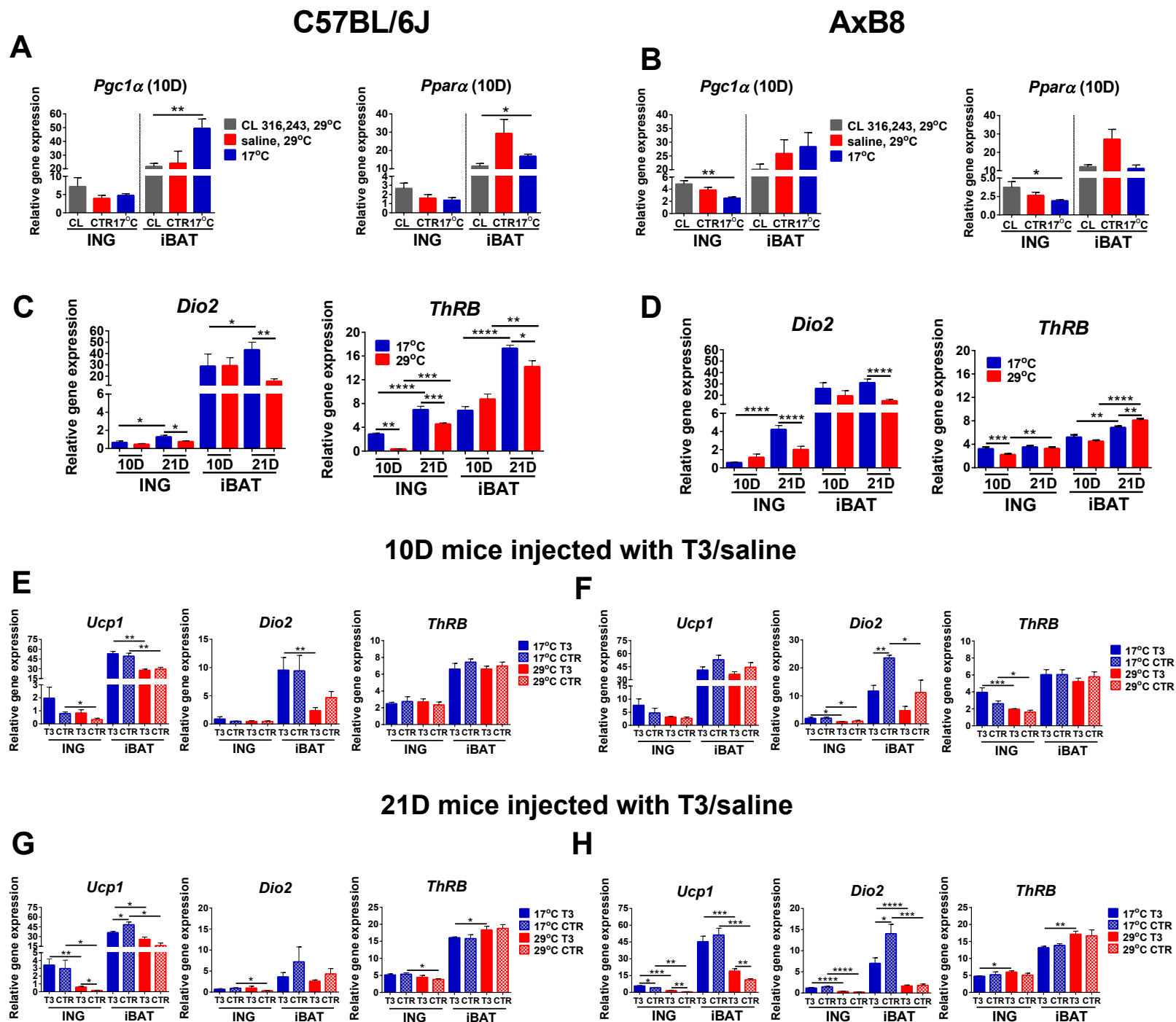

Supplement: Supplemental Data [file supp_fj.15-271395_Supplemental_Figure2.pdf]
