# Supplementary material for: Low ambient temperature during early postnatal development fails to cause a permanent induction of brown adipocytes
Source: FASEB J. 2015 Apr 20;29(8):3238–52. doi: 10.1096/fj.15-271395 (PMC4511198; doi:10.1096/fj.15-271395)
Supplement: Supplemental Data [file supp_fj.15-271395_Supplemental_Table1.pdf]

Table S1. Phenotypes of C57BL/6J and AxB8 mice maintained at different ambient temperatures during early post-natal development (related to Figure 1) - an effect of the ambient temperature or genetic background (Two-way ANOVA).

		C57BL/6J		AxB8		Two-way ANOVA		
		17°C	29°C	17°C	29°C			
Day		Mean (g) ± SD	Mean (g) ± SD	Mean (g) ± SD	Mean (g) ± SD	strain	temperature	interaction
7	Number of mice	**57**	**42**	**56**	**64**			
	Body weight	3.97 ± 0.53	3.82 ± 0.60	3.66 ± 0.57	3.76 ± 0.64	*	ns	no
	Fat mass	0.38 ± 0.13	0.45 ± 0.11	0.43 ± 0.16	0.52 ± 0.12	**	****	no
	Lean mass	3.30 ± 0.44	3.11 ± 0.49	2.92 ± 0.48	2.89 ± 0.60	****	ns	no
	Adiposity	0.12 ± 0.05	0.14 ± 0.02	0.14 ± 0.04	0.19 ± 0.06	****	****	no
10	Number of mice	**54**	**52**	**56**	**71**			
	Body weight	5.06 ± 0.68	5.01 ± 0.52	4.79 ± 0.70	4.73 ± 0.80	**	ns	no
	Fat mass	0.37 ± 0.17	0.50 ± 0.13	0.41 ± 0.13	0.59 ± 0.14	***	****	no
	Lean mass	4.16 ± 0.55	3.94 ± 0.45	3.74 ± 0.55	3.59 ± 0.65	****	*	no
	Adiposity	0.09 ± 0.04	0.13 ± 0.03	0.11 ± 0.03	0.17 ± 0.04	****	****	yes (*)
15	Number of mice	**47**	**48**	**46**	**46**			
	Body weight	6.65 ± 0.62	6.44 ± 0.63	5.75 ± 0.73	5.87 ± 0.79	****	ns	no
	Fat mass	0.31 ± 0.23	0.49 ± 0.16	0.44 ± 0.13	0.65 ± 0.17	****	****	no
	Lean mass	5.47± 0.62	5.14 ± 0.55	4.46 ± 0.64	4.49 ± 0.63	****	ns	no
	Adiposity	0.06 ± 0.04	0.10 ± 0.03	0.10 ± 0.02	0.15 ± 0.03	****	****	no
21	Number of mice	**51**	**48**	**48**	**51**			
	Body weight	7.55 ± 0.77	8.26 ± 1.17	7.10 ± 1.00	7.43 ± 1.10	****	***	no
	Fat mass	0.64 ± 0.29	0.91 ± 0.33	0.77 ± 0.16	0.89 ± 0.20	ns	****	yes (*)
	Lean mass	6.06 ± 0.77	6.55 ± 1.12	5.28 ± 0.88	5.72 ± 0.81	****	***	no
	Adiposity	0.10 ± 0.04	0.14± 0.03	0.15 ± 0.03	0.16 ± 0.03	****	***	yes (**)
56	Number of mice	**31**	**31**	**31**	**34**			
	Body weight	23.18 ± 1.19	21.98 ± 1.20	20.28 ± 1.56	20.09 ± 1.26	****	**	yes (*)
	Fat mass	3.68 ± 0.47	3.38 ± 0.33	3.15 ± 0.50	3.38± 0.43	***	ns	yes (**)
	Lean mass	17.87 ± 1.30	16.49 ± 1.41	16.02 ± 1.22	15.68 ± 1.05	***	****	yes(*)
	Adiposity	0.21 ± 0.02	0.21 ± 0.02	0.20 ± 0.03	0.22 ± 0.03	ns	*	yes (*)
112	Number of mice	**16**	**16**	**15**	**15**			
	Body weight	35.19 ± 2.83	38.52 ± 3.06	29.84 ± 3.31	32.79 ± 3.66	****	***	no
	Fat mass	10.52 ± 2.27	13.44 ± 2.19	7.46 ± 2.01	9.69 ± 2.44	****	****	no
	Lean mass	21.83 ± 0.77	22.18 ± 1.13	19.78 ± 1.29	20.41 ± 1.34	****	ns	no
	Adiposity	0.48 ± 0.10	0.60 ± 0.08	0.37 ± 0.09	0.47 ± 0.10	****	****	no

Data are mean ± SD. p<0.05; ** p<0.01; *** p<0.001; **** p<0.0001
