# Supplementary material for: Low ambient temperature during early postnatal development fails to cause a permanent induction of brown adipocytes
Source: FASEB J. 2015 Apr 20;29(8):3238–52. doi: 10.1096/fj.15-271395 (PMC4511198; doi:10.1096/fj.15-271395)
Supplement: Supplemental Data [file supp_fj.15-271395_Supplemental_Table2.pdf]

Table S2. Top genes up-regulated in inguinal WAT of 21D B6 mice as an effect of decreased ambient temperature (n=8 for both 17°C and 29°C) (related to Figure 3). Fold change difference is generated based on normalized signals (not shown).

Gene symbol	Gene name	P value (corr)	FC	Raw data 17°C	Raw data 29°C
**Cyp2b10**	Cytochrome P450, family 2, subfamily b, polypeptide 10	0.0042	16.69	2861.06	174.60
**Ucp1**	Uncoupling protein 1	0.0329	5.94	57633.08	10307.54
**Lrtm1**	Leucine-rich repeats and transmembrane domains 1	0.0104	4.09	447.93	108.70
**Cyp2f2**	Cytochrome P450, family 2, subfamily f, polypeptide 2	0.0187	2.56	25162.36	10328.67
**Aspg**	Asparaginase homolog (S. cervisiae)	0.0098	2.36	5494.65	2441.37
**Car14**	Carbonic anhydrase 14	0.0095	2.32	758.87	332.26
**Poln**	DNA polymerase N	0.0179	2.25	3336.37	1545.85
**Gyk**	Glycerol kinase	0.0273	2.25	535.15	241.20
**Acot11**	Acyl-coA thioesterase 11	0.0350	2.23	1200.45	551.28
**Trim67**	Tripartite motif-containing 67	0.0349	2.03	715.38	358.93
**Cox7a1**	Cytochrome c oxidase subunit VIIA 1	0.0485	1.87	82695.64	46334.86
**Gpd2**	Glycerol phosphatase dehydrogenase 2, mitochondrial	0.0180	1.84	3994.36	2272.89
**Slc25a35**	Solute carrier family, 25, member 35	0.0273	1.83	2559.27	1457.64
**Cldn5**	Claudin 5	0.0076	1.80	12119.40	7132.87
**C1qtnf4**	C1q and tumor necrosis factor related protein	0.0178	1.78	317.88	179.46
**Cox8b**	Cytochrome c oxidase subunit VIIIb	0.0122	1.69	147178.90	91980.50
**Zfp787**	Zinc finger protein 787	0.0043	1.68	3304.99	2047.76
**Slc26a10**	Solute carrier family, 26, member 10	0.0043	1.65	320.51	194.24
**Bcl6**	B cell leukemia/lymphoma 6	0.0471	1.65	12571.11	8073.23
**Mtor**	Mechanistic target of rapamycin (serine threonine kinase)	0.0319	1.61	361.11	226.37
**Cidea**	Cell death-inducing DNA fragmentation factor, alpha subunit-like effector A	0.0460	1.60	154086.10	102445.40
